# Preparation of Chitosan Molecularly Imprinted Polymers and the Recognition Mechanism for Adsorption of Alpha-Lipoic Acid

**DOI:** 10.3390/molecules25020312

**Published:** 2020-01-13

**Authors:** Long Xu, Ze-Xin Zhao, Yun-An Huang, Qiu-Jin Zhu

**Affiliations:** 1 School of Liquor and Food Engineering, Guizhou University, Guiyang 550025, China; xulong19891026@163.com; 2 School of Food Science and Engineering, South China University of Technology, Guangzhou 510640, China; 3 School of Bioscience and Bioengineering, South China University of Technology, Guangzhou 510006, China; bizexin-zhao@mail.scut.edu.cn; 4 Department of Food Engineering, Guizhou Vocational College of Foodstuff Engineering, Guiyang 551400, China; hya127@163.com; 5 Key Laboratory of Agricultural and Animal Products Store and Processing of Guizhou Province, Guiyang 550025, China

**Keywords:** alpha-lipoic acid, chitosan, molecular dynamics, molecularly imprinted polymers, molecular dynamics

## Abstract

Two effective molecularly imprinted polymers for the adsorption of alpha-lipoic acid (ALA) were synthesized by the cross-linking of chitosan with epichlorohydrin (ECH) and glutaraldehyde (GLU), respectively, in the presence of ALA as template molecules. Investigations on the molar ratios of ALA and chitosan (–NH_2_) in the preparation of chitosan molecularly imprinted polymers (MIPs) were carried out with a factor of ALA rebinding capabilities. The surface morphology and chemical properties of the polymers were characterized. The optimized MIPs crosslinked by ECH (MIPs–ECH) and MIPs crosslinked by GLU (MIPs–GLU) had adsorption capabilities of 12.09 mg/g and 19.72 mg/g for ALA, respectively. The adsorption behaviors of two kinds of chitosan MIPs including adsorption kinetics and isotherms were investigated in detail. Adsorption and kinetic binding experiments showed that the prepared MIPs–ECH and MIPs–GLU had selective adsorption and excellent affinity for ALA. In addition, the possible binding models between ALA and chitosan oligosaccharide were predicted by molecular dynamics simulation.

## 1. Introduction

The recognition of oxidative stress as an underlying factor in health, aging and disease has led to a surge in studies aimed at uncovering effective antioxidants. As a coenzyme in mitochondrial energy metabolism and cell redox modulator, alpha-lipoic acid (ALA) has gained considerable attention due to its potent biological antioxidant activity. In recent decades, mounting data have corroborated a broad spectrum of the potential health benefits of ALA including protection against cardiovascular disease [1], anti-inflammation [2], treatment for Alzheimer’s disease and related dementias [3] and the inhibition of tumorigenesis [4]. These positive protective and curative effects have fostered significant motives in the chemical and enzymatical synthesis of ALA [5,6,7,8,9,10,11,12,13,14,15]. However, the synthesis is generally involved with expensive starting materials, complicated steps and low overall product yields [16].

Inevitably, the safety issues of the applications of chemically synthesized ALA in food, cosmetics and medicine become a great concern for consumers. Therefore, the isolation and enrichment of naturally occurring ALA from animal and plant tissues both serve as a promising alternative to obtain this powerful antioxidant. In the best of previous studies, an effective protocol for ALA extraction from natural products can be hardly found. Gratifyingly, molecular imprinting technology (MIT) provides a great potential for the isolation and enrichment of natural ALA.

Recent decades have witnessed tremendous developments in MIT, which is a useful technique to construct specific sites for the target compounds (template) in the preparation of molecularly imprinted polymers (MIPs). These polymers have attracted increasing attentions due to their considerable advantages, including predetermined recognition ability, robustness, low cost, easy synthesis, excellent reusability and wide applications [17]. The most common preparation of MIPs is based upon the copolymerization of functional monomers and crosslinkers in the presence of template molecules. After the template molecules being eluted, the formed cavities are specifically complementary to the template molecules in shape, size and chemical functionality, thereby facilitating a selective recognition of template molecules. These characteristics have contributed to extensive applications of MIPs in the extraction of natural products [18,19,20,21].

As a vital element affecting the recognition selectivity of MIPs, a suitable functional monomer generally contains several functional groups that can strongly interact with the template and form specific donor–receptor complexes. Some versatile monomers (e.g., methacrylic acid, acrylic acid, acrylamide, etc.) for molecular imprinting, are well-documented in previous critical reviews [17,22]. Also, a natural polyaminosaccharide, chitosan, has been well recognized as a promising alternative of functional monomers due to the abundant amino and hydroxyl groups on its polysaccharide chain [23,24]. These functional groups enabled the flexible preparation of MIPs for water pollution control [25,26], controlled drug delivery [27] and chiral resolution [28,29]. The crosslinking mechanism of molecular imprinting technology with chitosan as functional monomers is described in detail in a previous publication [23]. However, a comprehensive investigation of intermolecular interactions between chitosan oligosaccharide and template molecules in chitosan MIPs is still very limited [30].

In the present study, two chitosan MIPs were synthesized with epichlorohydrin (ECH) and glutaraldehyde (GLU) as crosslinkers, and used for the adsorption of ALA. The surface morphology and chemical properties of these polymers were characterized. The adsorption capacity and selectivity of ALA were determined and compared. Additionally, the intermolecular interactions of chitosan oligosaccharide and ALA were investigated by molecular dynamics. We believe that this theoretical study on the recognition mechanism will contribute greatly to the application of chitosan MIPs in the isolation and enrichment of ALA from natural resources.

## 2. Results and Discussion

### 2.1. The Molar Ratio of ALA to Chitosan

**Figure 1:**
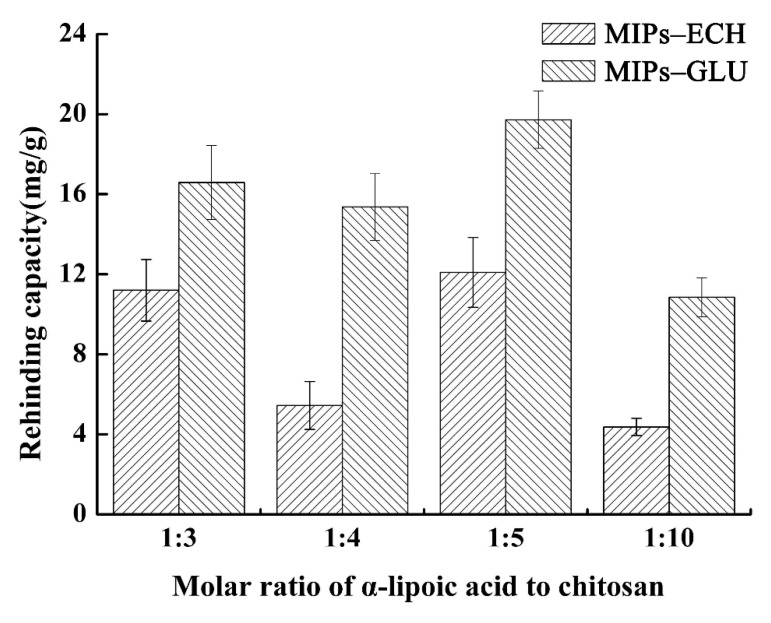
Effect of different molar ratio of alpha-lipoic acid (ALA) to chitosan on the rebinding efficiency of molecularly imprinted polymers (MIPs).

Admittedly, the molar ratio of the template to the functional monomer is a vital parameter affecting greatly on the rebinding capacities and imprinting efficiency of MIPs. Less available recognition sites can be formed with insufficient templates during polymerization. In contrast, excessive templates may lead to the deficiency of MIPs due to the relative lack of functional groups [31]. The rebinding capacities of MIPs–ECH and MIPs–GLU were investigated, ranging from 1:3 to 1:10 (Figure 1). As it can be seen in Figure 1, the rebinding capacities of MIPs increased with the increment of the ALA/chitosan (–NH_2_) ratio. The peak rebinding capacities of 12.09 mg/g and 19.72 mg/g could be observed at a same ratio of 1:5 with respect to MIPs–ECH and MIPs–GLU. Further increment of the molar ratio, however, resulted in declined rebinding capacities. A consistent observation was also demonstrated by previous studies [31]. Moreover, it was observed that the rebinding capacity of MIPs–GLU was more than that of MIPs–ECH.

### 2.2. Characterization of MIPs

**Figure 2:**
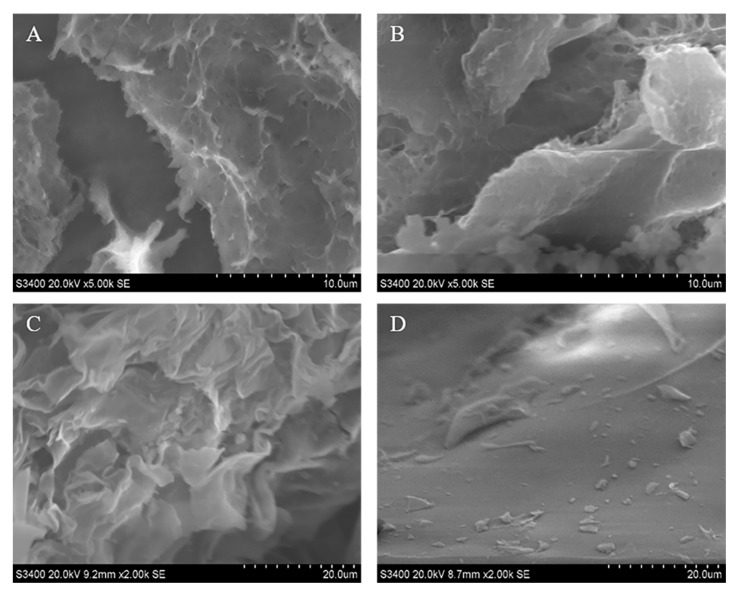
Scanning electron micrographs of (**A**) MIPs–ECH, (**B**) NIPs–ECH, (**C**) MIPs–GLU and (**D**) NIPs–GLU.

Investigated by scanning electron microscope, the morphological differences of prepared MIPs–ECH and NIPs–ECH, MIPs–GLU and NIPs–GLU are presented in Figure 2. It could be observed that compared with NIPs–ECH, MIPs–ECH displayed a rougher surface with many micropores. Presumably, this structural feature is attributed to the cavities left after the elution of ALA from the cross-linked network. Morphologically, MIPs–GLU were filled with wavy wrinkles. This featured spatial structure is also probably associated with the adsorption capabilities. By contrast, NIPs–GLU showed a relatively smooth surface.

**Figure 3:**
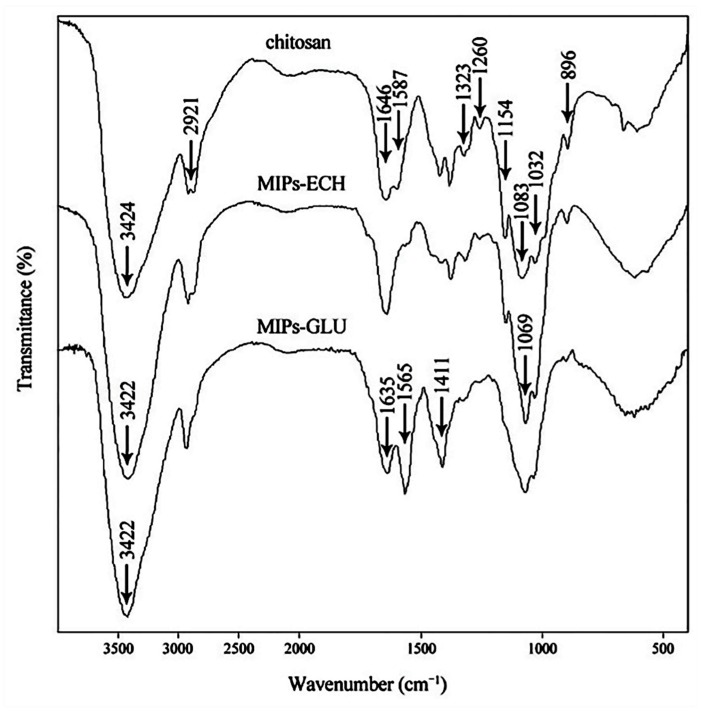
Fourier transform infrared (FTIR) spectra of chitosan, MIPs–ECH and MIPs–GLU.

The FTIR spectra of chitosan, MIPs–ECH and MIPs–GLU are shown in Figure 3. The spectrum of pure chitosan exhibited the characteristic peaks at about 896 and 1154 cm^−1^, corresponding to saccharide structure. The adsorption peaks at 1646, 1587 and 1323 cm^−1^ are the characteristic of amides I, II and III, respectively. The sharp peaks at 1383 and 1422 cm^−1^ were assigned to the –CH_3_ symmetrical deformation mode. The adsorption peak at 1260 cm^−1^ was the adsorption of δ(O–H). The broad peaks at 1032 and 1083 cm^−1^ were caused by the C–O stretching vibration in chitosan. Another broad peak at 3424 cm^−1^ was due to amine N–H symmetrical vibration, which was generally used for the quantitative analysis of the deacetylation of chitosan with 1646 cm^−1^. The peak at 2921 cm^−1^ reflected the typical C–H stretch vibrations. With respect to MIPs–ECH, the adsorption peak corresponding to the stretching vibration of N–H and O–H shifted slightly to lower wavenumbers, which was caused by the hydrogen bond strength. Another broad adsorption peak at about 1200–900 cm^−1^ corresponded to the C–O structure generated by the cross-linking reaction between ECH and chitosan. With respect to MIPs–GLU, the bending vibration peak of CH_2_ intensified and shifted to 1411 cm^−1^. 1635 cm^−1^ and 1565 cm^−1^ corresponded to the C=N and NH_2_ groups. The adsorption peak of C=N strengthened obviously, whereas the adsorption peak of –NH_2_ weakened distinctly. It demonstrated that the Schiff base formed between the amino groups of chitosan and the aldehyde groups of glutaraldehyde. The adsorption peak also shifted to lower wavenumbers, demonstrating the hydrogen bond formed between ALA and chitosan. Collectively, the above changes of adsorption peaks proved that ALA has been successfully imprinted in chitosan, possibly via hydrogen bonds between –NH_2_ and –OH on chitosan chains and –OH in the template molecule [32].

### 2.3. Binding Properties of the MIPs and NIPs

#### 2.3.1. Kinetic Adsorption

**Figure 4:**
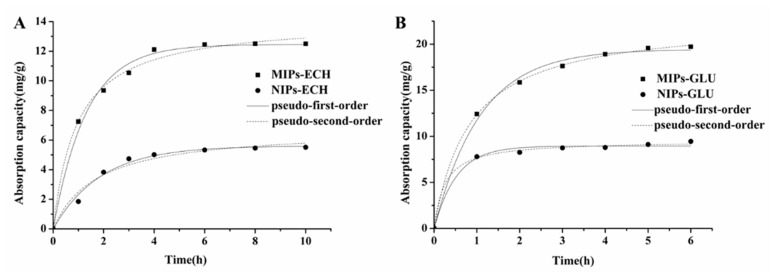
Adsorption kinetics of alpha-lipoic acid (ALA) on molecularly imprinted polymers (MIPs) and non-imprinted polymers (NIPs). (**A**) MIPs–ECH and NIPs–ECH, (**B**) MIPs–GLU and NIPs–GLU.

The adsorption kinetics of MIPs–ECH and NIPs–ECH, MIPs–GLU and NIPs–GLU for ALA are shown in Figure 4. The ALA binding capacity of MIPs was much higher than that of NIPs. Additionally, the adsorption behaviors of MIPs and NIPs were time-dependent. Different kinetic profiles for ALA could be clearly observed.

With respect to MIPs–ECH, before reaching the binding equilibrium at 6 h, a high increasing rate at the beginning of adsorption was observed in the first 4 h. However, the adsorption rate of NIPs–ECH increased almost linearly before the first 2 h. Then it reached the equilibrium after 4 h. Possibly, the numerous micropores on the MIPs–ECH surface resulted in the fact that it took more time for ALA to diffuse into MIPs–ECH before reaching equilibrium. The adsorption capacities of MIPs–ECH reached 12.50 mg/g compared with 5.52 mg/g of NIPs–ECH.

With respect to the adsorption kinetics of MIPs–GLU, the amount of ALA absorbed by MIPs–GLU increased rapidly in the first 3 h. A subsequent gradual increasing rate was observed before the equilibrium at 5 h. By comparison, NIPs–GLU reached the adsorption equilibrium within only about 2 h. The maximal adsorption capacities of MIPs–GLU adsorbents for ALA reached 19.72 mg/g, which was significantly higher than the 9.45 mg/g of NIPs–GLU adsorbents. The differences of adsorption capacities and kinetics are probably attributed to the special 3D spatial structures of MIPs with high affinities.

Compared with a previous study [33], the chitosan MIPs–ECH reported herein displayed parallel ALA adsorption capabilities, whereas MIPs–GLU showed 1.6-fold increase in ALA adsorption capabilities. Overall, both MIPs–ECH and MIPs–GLU showed better imprinting effects than NIPs–ECH and NIPs–GLU, respectively.

The Lagergren pseudo-first-order and pseudo-second-order models [34,35] are usually used for the description of the adsorption kinetics. The differential equation of the pseudo-first-order model is as follows:

(1) $$\frac{dQ}{dt}=k_{1}\left(Q_{e}-Q\right)$$

The above non-linear equation can be converted into the following Equation:

(2) $$\mathrm{lg}\left(Q_{e}-Q_{t}\right)=\mathrm{lg}Q_{e}-\frac{k_{1}}{2.303}t$$

*Q_e_* represents the amount of ALA absorbed at equilibrium, mg/g; *Q_t_* is the amount of ALA absorbed at time *t*, mg/g; *k*_1_ is the equilibrium rate constant of pseudo-first adsorption, min^−1^.

The pseudo-second-order model is based upon the assumption that the adsorption behavior is controlled by a chemical adsorption mechanism, which involves electron sharing or electron transfer. The differential equation of the pseudo-second-order model is as follows:

(3) $$\frac{dQ}{dt}=k_{2}{\left(Q_{e}-Q\right)}^{2}$$

Equation (3) can be rearranged to obtain a linear form:

(4) $$\frac{t}{Q_{t}}=\frac{1}{k_{2}Q_{e}^{2}}+\frac{1}{Q_{e}}t$$

*Q_e_* is the amount of ALA absorbed at equilibrium, mg/g; *Q_t_* is the amount of ALA absorbed at time *t*, mg/g; *k*_1_ is the equilibrium rate constant of pseudo-first adsorption, g mg^−1^ min^−1^.

As shown in Figure 4 and Table 1, the pseudo-second-order model fitted the experimental data better than the pseudo-first-order model based on the correlation coefficient (*r*^2^). The good fit (*r*^2^ > 0.99) obtained via the second-order model demonstrated that the adsorption of ALA onto the MIPs–ECH and MIPs–GLU conformed to the chemical reaction mechanism. The above observation demonstrated that the adsorption behavior was dominated by chemical adsorption [25].

#### 2.3.2. Static Adsorption

**Figure 5:**
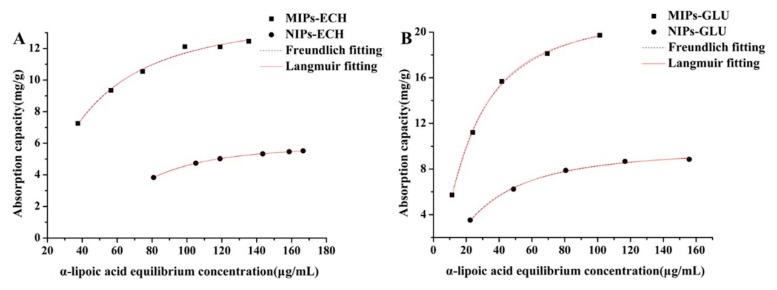
Adsorption isotherms of the (**A**) MIPs–ECH and NIPs–ECH, (**B**) MIPs–GLU and NIPs–GLU for ALA at 30 °C.

In order to investigate the affinity of MIPs and NIPs for ALA, the binding experiments and following Scatchard analysis were performed in an initial ALA concentration range of 0.04–0.2 mg/mL. As shown in Figure 5, the adsorption capacity increased with the initial concentration increment of ALA. The results also showed that the amount of ALA absorbed by MIPs was significantly higher than NIPs, indicating numerous specific ALA binding sites formed during the imprinting reaction.

### 2.4. Adsorption Isotherms

Adsorption isotherms were tested at a constant temperature with different initial concentrations of ALA. The effects of the initial ALA concentration on the adsorption capacities of MIPs and NIPs at 30 °C are illustrated in Figure 5. The isotherm curves demonstrated that the equilibrium adsorption capacities of both MIPs and NIPs increased with the increment of initial ALA concentrations. Obviously, MIPs showed much higher adsorption amounts than NIPs within all tested solution concentrations. Therefore, specific molecular recognition sites were successfully formed in MIPs for the template molecule ALA during the polymerization process.

To further investigate the imprinting effect, the binding properties of isotherms were estimated according to Langmuir and Freundlich models to establish the adsorption system.

The Langmuir model is as follows:

(5) $$\frac{C}{Q}=\frac{1}{Q_{\max}K_{L}}+\frac{C}{Q_{\max}}$$

The Freundlich model is as follows:

(6) $$\log Q=\frac{\log C}{n}+\log K_{F}$$

where *C* is the equilibrium concentration of ALA (mg/mL), *Q* is the adsorption capacity of MIP at equilibrium concentration (mg/g), *Q*_max_ is the maximum adsorption capacity (mg/g), *K_L_* is the Langmuir constant (g/mL), *n* and *K_F_* are Freundlich constants (g/mL).

Figure 5 illustrated the fitting results of Langmuir and Freundlich equations on the adsorption isotherms of the MIPs–ECH and NIPs–ECH, MIPs–GLU and NIPs–GLU for ALA. It was noted that both the Langmuir and Freundlich equations fit all the data well.

It is well-documented that many real adsorption processes of monolayer adsorption conform to the Langmuir adsorption isotherm [36]. This observation is based on an assumption that all adsorption sites in a structurally homogeneous adsorbent are identical and equivalent energetically. According to the Langmuir isotherm equation, once a molecular occupies a site, further adsorption cannot take place at the same site. Probably, the good fit for the Langmuir equation was indicative of a predominant chemical adsorption. Also, it served as a strong indicator of monolayer adsorption on the surface of MIPs. Further adsorption behavior could not be observed, as the specific cavities in MIPs for ALA reached “a saturated state”. Moreover, it is demonstrated that the Langmuir constants (*K_L_*) of MIPs–ECH and MIPs–GLU were higher than that of NIPs–ECH and NIPs–GLU, respectively. indicating stronger affinity of the MIPs–ECH and MIPs–GLU for the ALA template.

The Freundlich equation is an empirical equation applied to describe heterogeneous systems, in which it is characterized by the heterogeneity factor 1/*n* [36].

The Freundlich equation also fitted the experimental data well, which demonstrated that the adsorption process depended upon a noncovalent interaction.

### 2.5. Scatchard Analysis

Scatchard proposed a plotting method, aiming at analysis of the binding relation of ions, drugs and other molecules with protein (including receptors). The Scatchard equation is as follows:

(7) $$\frac{Q}{Q_{s}}=\frac{Q_{\max}-Q}{K_{d}}$$

For the receptors binding experiment, *Q* is the concentration of receptors, *Q*_s_ is the concentration of free ligands, *K_d_* is the dissociation constant and *Q*_max_ is maximal saturation concentration of ligand binding sites.

**Figure 6:**
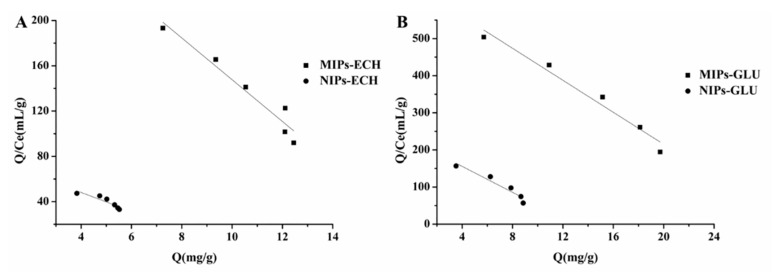
Scatchard plot analysis of the binding property of ALA to MIPs and NIPs. (**A**) MIPs–ECH and NIPs–ECH, (**B**) MIPs–GLU and NIPs–GLU.

The Scatchard equation is also usually applied to analyze the saturation binding data of the template onto MIPs and NIPs, which can further estimate the binding properties. It can be seen in Figure 6 that there are two straight lines obtained in the case of MIPs in the plot region, which indicates that only one type of binding sites exists in MIPs–ECH and MIPs–GLU. For MIPs–ECH and NIPs–ECH, the linear regression equations were *Q*/*Q*_s_ = 332.17 − 18.45*Q* and *Q*/*Q*_s_ = 81.63 − 8.37*Q*, respectively. The dissociation constant (*K_d_*) of 0.05 and 0.12 mg/mL, and the apparent maximum binding capacities (*Q*_max_) of 18.00 and 9.75 mg/g could be calculated from the slopes and the intercept of the linear equilibrations. In the case of MIPs–GLU and NIPs–GLU, the linear regression equations were *Q*/*Q*_s_ = 646.32 − 21.56*Q* and *Q*/*Q*_s_ = 225.71 − 17.49*Q*, respectively. The dissociation constant (*K_d_*) of 0.05 and 0.06 mg/mL, and the apparent maximum binding capacities (*Q*_max_) of 29.98 and 12.91 mg/g. The specific affinity and binding capacity of MIPs–ECH and MIPs–GLU for ALA were much higher than that of NIPs–ECH and NIPs–GLU, respectively.

### 2.6. MD Simulation

**Figure 7:**
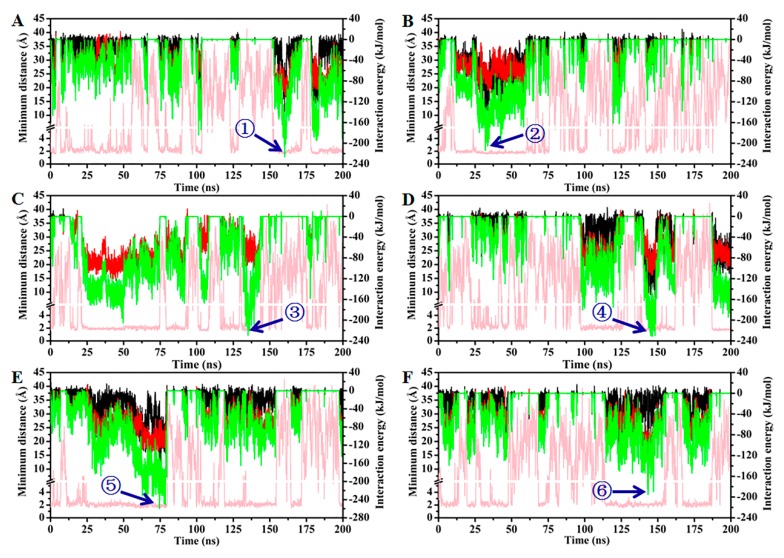
The computational results analysis (**A**–**F**) The minimum distances (pink line), vdW (black line), Coulomp (red line) and total (green line) interaction energy between ALA 1–6 and chitosan, respectively.

**Figure 8:**
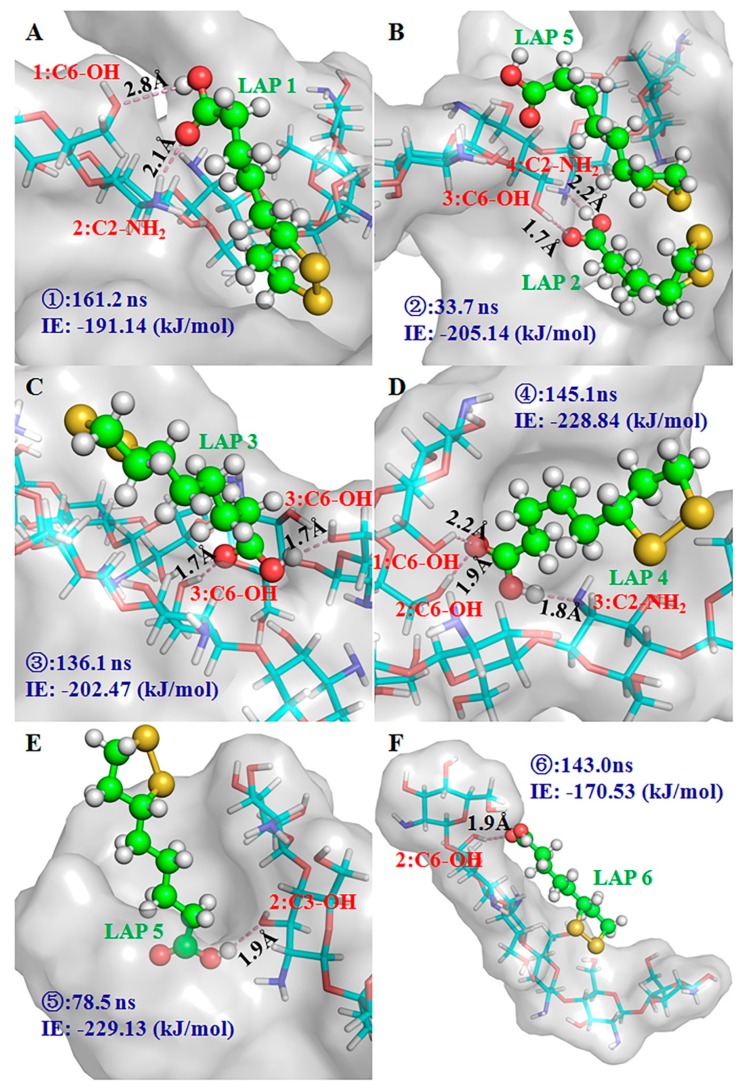
The conformation in the highest interaction energy snapshots. (**A**–**F**) ALA 1–6 are shown as a ball-and-stick model and are labeled in green; the groups of chitosan which participated in the formation of the hydrogen bond are labeled in red (unit number: carbon number-group). The chitosan presented as the gray surface and the hydrogen bonds were showed as a pink dash. The simulation time and interaction energy (IE) were labeled in blue.

MD simulation serves as a powerful tool to illuminate the molecular interaction between functional monomers and template molecules in MIPs. To further investigate the atomic-level dynamic and interaction information of the MIP system, an 8 × 8 × 8 Å periodic water box with five chitosan hexamers and six ALA molecules was established for performing a 200 ns MD simulation study (Figure S1). The spontaneous aggregation of chitosan chains was observed after a short simulation time (Figure S2A). However, the tremendous fluctuation of the root mean square deviation (RMSD) indicated that the system did not form a stable conformation (Figure S2B). Presumably, a lack of covalence connection of the crosslinker led to the fluctuation. Although a real system could not be mimicked completely, some interesting binding conformations between chitosan chains and ALA molecules were observed in the simulation process. The MD simulation displayed the binding model of ALA in the cavity of the chitosan micelle. As shown in Figure 7, the minimum distances between ALAs (numbering from 1 to 6) and chitosan were less than 3 Å at some points in the simulation. It is noteworthy that the interaction energy (IE) between ALAs and chitosan was various at these time points. It is generally acknowledged that higher IE values are indicative of a more stable binding conformation. Thus, snapshots with the strongest IE between each ALA and chitosan in the simulation were selected to further analyze the conformations. Two hydrogen bonds were formed in the most stable binding conformation of ALA 1, 2 and 3 and ALA molecules in the superficial groove formed by the aggregation of chitosan chains. All IEs between chitosan and these ALAs were ~200 kJ/mol. The detailed binding conformations were illustrated in Figure 8A–C. The snapshot at 33.7 ns showed that the groove could also accommodate ALA 5 without hydrogen bond interaction in addition to ALA 2 (Figure 8B). The IE of ALA 5 with chitosan was 66.77 kJ/mol, which was lower than the IE of ALA 2 with chitosan. Therefore, hydrogen bond interactions greatly contributed to the absorption stability of ALA molecules. The highest interaction energy of ALA 4 and 5 with chitosan were 228.84 and 229.12 kJ/mol, respectively. Three hydrogen bonds were formed between ALA 4 and the chitosan micelle in the strong interaction state, while ALA 5 only generated one hydrogen bond with the hydroxyl group located in the C3 atom of the second unit of a chitosan hexamer (Figure 8D,E). In the binding conformation of ALA 4 and 5 described above, the polar heads of both ALA molecules plugged into the deep cavities instead of lying in the superficial groove like ALA 1, 2 and 3. Although the number of hydrogen bonds of ALA 4 and 5 with chitosan was different, a comparable level was observed regarding their IE values. Therefore, the surface geometry of the chitosan micelle might be a critical factor in determining the capture efficiency of ALA molecules. Based on these observations, it could be concluded that various crosslinkers used in the preparation of chitosan MIPs greatly affected the geometry configuration of chitosan, thereby resulting in the different absorption capabilities of real MIP systems. With regard to ALA 6, the snapshot in the highest IE value (–170.53 kJ/mol, Figure 8F) showed that this molecule was surrounded by only one chitosan hexamer. As a result, an effective absorption behavior could not be observed.

## 3. Materials and Methods

### 3.1. Chemicals

Chitosan with a deacetylation degree of 90% was purchased from the Beijing Biotopped Science and Technology Co., Ltd. (Beijing, China). Epichlorohydrin and alpha-lipoic acid were obtained from Sigma-Aldrich (St. Louis, MO, USA). Glutaraldehyde, liquid paraffin and formaldehyde (AR, 37.0 *w*/*v*%) were supplied by Chengdu Jinshan Chemical Reagent Co., Ltd. (Chengdu, China). Span-80 was obtained from Tianjin Fuchen Chemical Co., Ltd. (Tianjin, China). Methanol and tetrachloromethane were of analytical grade and supplied by Tianjin Kermel Chemical Reagent Development Center (Tianjin, China). 

### 3.2. Preparation of Chitosan MIPs

#### 3.2.1. ECH as Cross-Linker

Chitosan was cross-linked with epichlorohydrin (ECH) and molecularly imprinted with alpha-lipoic acid (ALA) as the template molecule (MIPs–ECH) following a single-step procedure as previously described with modifications [31]. Firstly, 0.48 g of chitosan was dissolved in 24 mL of 2% acetic acid aqueous solution (*v*/*v*). The resulted chitosan solution was mixed with 20 mL of methanol containing 112 mg of ALA (1:5, the molar ratio of ALA to –NH_2_ groups in chitosan) and the mixture was stirred for 30 min. 48 mL of liquid paraffin containing 0.4 mL of span-80 were added into the mixture solution and stirred at 50 °C for 10 min. Then, 0.5 mL of formaldehyde aqueous solution (37%, *w*/*w*) performed as amino protective solute was added into the mixture and stirred for 30 min. After the pH was adjusted to 9.0 with 2 M sodium hydroxide (NaOH) aqueous solution, 620 μL of ECH was added dropwise into the mixture. The cross-linking reaction was performed by agitation at 70 °C for 3 h with the pH maintained at 9.0 throughout. To remove the paraffin and span-80, the resulting molecularly imprinted polymers (MIPs) were washed by excess petroleum ether. Then, the petroleum ether residues were removed by methanol. Hydrochloric acid (HCl) (0.1%) was used to remove the amino protective solute.

To elute the template molecules, MIPs–ECH were extracted in a Soxhlet apparatus with methanol/acetic acid (9:1, *v*/*v*) for 72 h. Subsequently, these MIPs–ECH were washed with excess deionized water and then dried in a vacuum freeze dryer. Finally, the freeze-dried MIPs–ECH were ground in a mortar and sieved to get particles with a size about 74 μm and kept in a desiccator until use. Non-imprinted polymers (NIPs–ECH) were synthesized with the same procedure in the absence of the ALA template.

#### 3.2.2. GLU as Cross-Linker

Chitosan was cross-linked with GLU and molecularly imprinted with ALA (MIPs–GLU) as follows: Firstly, 0.48 g of chitosan was dissolved in 24 mL of 2% acetic acid aqueous solution (*v*/*v*). The resulted chitosan solution was mixed with 20 mL of methanol containing 112 mg of ALA (1:5, the molar ratio of ALA to the –NH_2_ group in chitosan) and the mixture was stirred for 30 min. 500 μL of glutaraldehyde (GLU) was added dropwise into the mixture. The cross-linking reaction was performed by agitation at 25°C for 12 h.

To elute the template molecules, MIPs–GLU were extracted in a Soxhlet apparatus with methanol/acetic acid (9:1, *v*/*v*) for 72 h. Finally, MIPs–GLU were washed with excess deionized water and then dried in a vacuum freeze dryer. Finally, the freeze-dried MIPs–GLU were ground in a mortar and sieved to get particles with a size about 74 μm and stored in a desiccator until use. Non-imprinted polymers (NIPs–GLU) were synthesized with the same procedure in the absence of the ALA template.

### 3.3. Optimization of Molar Ratio of ALA to Chitosan for MIPs Preparation

The optimum molar ratios of the template (ALA) to the functional monomer (chitosan) ranging from 1:10, 1:5, 1:4 and 1:3 were tested for MIPs–ECH and MIPs–GLU preparation according to the rebinding capacities. All the other conditions were as formerly described.

### 3.4. Characterization of MIPs and NIPs

The synthesized MIPs and NIPs were characterized with scanning electron microscope (SEM) (S-3400, Hitachi Ltd., Tokyo, Japan) and Fourier transform infrared spectroscopy (FTIR, Vector 22, Bruker, Germany).

### 3.5. Kinetic Adsorption

In the kinetic experiments, 20 mg of MIPs were placed in a 10 mL tube and mixed with 5 mL of ALA (200 µg/mL) dissolved in tetrachloromethane. The suspensions were incubated for 12 h at 30 °C. Samples were collected at fixed intervals (1 h), filtrated with a 0.22 µm filter, and then analyzed by a Thermo Scientific Microplate Reader at 260 nm. The same experiments were performed for the corresponding NIPs.

The effect of initial ALA concentration on the adsorption was determined by the mixing of MIPs with 5 mL of ALA solutions at definite concentrations (40–200 µg/mL). The residual concentration of ALA was determined after adsorption. The adsorption capacity was calculated based on the following formula [37]:

(8) $$Q=\frac{\left(C_{i}-C_{f}\right)\times V}{m}$$

*Q* (mg/g) was the mass of ALA adsorbed per gram of MIPs, *C_i_* (µg/mL) was the initial concentration of ALA, *C_f_* (µg/mL) was the final concentration after adsorption, *V* (mL) was the total volume of adsorption mixture, and *m* (mg) was the mass of MIPs. The same experiments were performed for the respective NIPs.

The specific recognition characteristic of each MIP is defined as the imprinting factor (IF). It is calculated according to the following formula:

(9) $$IF=\frac{Q_{MIPs}}{Q_{NIPs}}$$

where *Q_MIPs_* and *Q_NIPs_* are the adsorption capacities of the MIPs and NIPs, respectively.

### 3.6. Computational Methods

The initial structure of the chitosan hexamer was obtained from the GLYCAM website (www.glycam.org). After the chitin β-(1→4)-2-amino-2-deoxy-d-glucopyranose hexamer was minimized according to the GLYCAM06 parameters [38], the acetyl group was deleted to obtain the chitosan hexamer. Meanwhile, the force field parameters of the chitosan hexamer and ALA molecule were generated from the AMBER GAFF force field [39]. Their partial atomic charges were obtained from the restrained electrostatic potential (RESP) charge at the HF/6–31G (d) level with the Gaussian 09 package [40].

Five chitosan hexamers and six ALA molecules were centrally placed into an 8 × 8 × 8 Å period water box (the distance of the buffer between the box wall and the nearest solute atom was more than 1.5 nm). The water molecules in the following simulation were used according to the TIP3P model [41].

The initial model was first minimized to relax the solvent and to optimize the system. After several steps of minimization, each model was heated to 300 K under the number–volume–temperature (NVT) ensemble for 100 ps, followed by another 100 ps of MD simulation under the number–pressure–temperature (NPT) ensemble to relax the system density to about 1.0 g/cm^3^ with a target temperature of 300 K and a target pressure of 1.0 atm. Subsequently, with a target temperature of 300 K and a time step of 2.0 fs, 200 ns of NPT MD simulation under periodic boundary conditions was performed for the prepared system to produce the trajectory by Gromac 5.1.7 [42]. Throughout the simulation process, the LINCS algorithm was applied to constrain all bonds. The Velocite-rescale and Parrinello–Rahman methods were used to control the system temperature and pressure, and a cutoff of 14 Å was set for both van der Waals and electrostatic interactions.

## 4. Conclusions

In the present study, two kinds of MIPs (MIPs–ECH and MIPs–GLU) were prepared based on two different cross-linkers (ECH and GLU) with chitosan as functional monomer and ALA as the template molecule. Both MIPs–ECH and MIPs–GLU exhibited better recognition to ALA than NIPs–ECH and NIPs–GLU, respectively. Further investigation on static adsorption and adsorption isotherms confirmed that the adsorption of ALA on MIPs–ECH and MIPs–GLU conformed to chemical adsorption. Scatchard analysis demonstrated that only one type of binding sites existed in the structure of MIPs–ECH and MIPs–GLU. MD simulation results demonstrated that the binding stability between chitosan and ALA was greatly affected by hydrogen bonds. More importantly, the surface geometry of the chitosan micelle, which were influenced by the crosslinkers, greatly determined the capture capability of the ALA molecules. In addition, a clear binding model between functional monomers and template molecules could contribute to the design of promising effective chitosan MIPs with better selectivity and higher adsorption capabilities. Overall, the chitosan MIPs will act as a promising sustainable absorbing material for the isolation and enrichment of ALA from natural resources.

## Figures and Tables

**Table 1:** Kinetic parameters of the pseudo-first-order and pseudo-second-order equations for ALA adsorption onto the MIPs and NIPs adsorbents.

Polymers	*Q_e_*(mg/g)	Pseudo-First-Order		Pseudo-Second-Order	
		*k*_1_	*R* ^2^	*k*_2_	*R* ^2^
MIPs–ECH	12.50	0.9200	0.9670	0.1079	0.9973
NIPs–ECH	5.50	0.5698	0.9933	0.1297	0.9786
MIPs–GLU	19.72	0.9141	0.9631	0.0703	0.9995
NIPs–GLU	9.45	0.5134	0.8617	0.3145	0.9979

## Supplementary Materials

The following are available online, Figure S1: The initial model of ALAs (ball-and-stick model) and chitosan hexamers (vdW surface model) in water box; Figure S2: RMSD analysis.

## Abbreviations

ALA	alpha-lipoic acid
ECH	epichlorohydrin
FTIR	Fourier transform infrared spectroscopy
GLU	glutaraldehyde
MD	molecular dynamics
MIT	molecular imprinting technology
MIPs	molecularly imprinted polymers
MIPs–ECH	molecularly imprinted polymers crosslinked by epichlorohydrin
MIPs–GLU	molecularly imprinted polymers crosslinked by glutaraldehyde
NIPs–ECH	non-imprinted polymers crosslinked by epichlorohydrin
NIPs–GLU	non-imprinted polymers crosslinked by glutaraldehyde
RESP	restrained electrostatic potential
RMSD	root mean square deviation

## References

[1] Wollin S.D., Jones P.J.H. (2003). α-lipoic acid and cardiovascular disease. J. Nutr..

[2] Zhang W.-J., Wei H., Hagen T., Frei B. (2007). α-Lipoic acid attenuates LPS-induced inflammatory responses by activating the phosphoinositide 3-kinase/Akt signaling pathway. Proc. Natl. Acad. Sci. USA.

[3] Holmquist L., Stuchbury G., Berbaum K., Muscat S., Young S., Hager K., Engel J., Münch G. (2007). Lipoic acid as a novel treatment for Alzheimer’s disease and related dementias. Pharmacol. Ther..

[4] Farhat D., Lincet H. (2020). Lipoic acid a multi-level molecular inhibitor of tumorigenesis. BBA-Rev. Cancer.

[5] TeresaáBes M., Peter W. (1995). Application of enzymic Baeyer-Villiger oxidations of 2-substituted cycloalkanones to the total synthesis of (*R*)-(+)-lipoic acid. J. Chem. Soc. Chem. Commun..

[6] Adger B., Bes M.T., Grogan G., McCague R., Pedragosa-Moreau S., Roberts S.M., Villa R., Wan P.W., Willets A.J. (1997). The synthesis of (*R*)-(+)-lipoic acid using a monooxygenase-catalysed biotransformation as the key step. Biorg. Med. Chem..

[7] Fadnavis N., Babu R.L., Vadivel S.K., Deshpande A.A., Bhalerao U. (1998). Lipase catalyzed regio- and stereospecific hydrolysis: Chemoenzymatic synthesis of both (*R*)-and (*S*)-enantiomers of α-lipoic acid. Tetrahedron Asymmetry.

[8] Zhang S., Chen X., Zhang J., Wang W., Duan W. (2008). An enantioselective formal synthesis of (+)-(*R*)-α-lipoic acid by an L-proline-catalyzed aldol reaction. Synthesis.

[9] Panchgalle S.P., Jogdand G.F., Chavan S.P., Kalkote U.R. (2010). Enantioselective synthesis of (*R*)-(+)-α-lipoic acid via proline-catalyzed sequential alpha-aminoxylation and HWE olefination of aldehyde. Tetrahedron Lett..

[10] Chavan S.P., Pawar K.P., Praveen C., Patil N.B. (2015). Chirality induction and chiron approaches to enantioselective total synthesis of alpha-lipoic acid. Tetrahedron.

[11] Olbrich M., Gewald R. (2006). Process for the Enantioselective Reduction of 8-Chloro-6-Oxo-Octanoic Acid Alkyl Esters. U.S. Patent.

[12] Müller M., Sauer W., Laban G. (2007). Method for the Production of (R)-and (S)-8-Chloro-6-Hydroxyoctanic Acid Alkyl Esters by Enzymatic Reduction. U.S. Patent.

[13] Gopalan A.S., Jacobs H.K. (1990). Bakers’ yeast reduction of alkyl 6-chloro-3-oxohexanoates: Synthesis of (*R*)-(+)-α-lipoic acid. J. Chem. Soc. Perkin Trans. 1.

[14] Zhou W.-J., Ni Y., Zheng G.-W., Chen H.-H., Zhu Z.-R., Xu J.-H. (2014). Enzymatic resolution of a chiral chlorohydrin precursor for (*R*)-α-lipoic acid synthesis via lipase catalyzed enantioselective transacylation with vinyl acetate. J. Mol. Catal. B Enzym..

[15] Zhang Y.-J., Zhang W.-X., Zheng G.-W., Xu J.-H. (2015). Identification of an ε-keto ester reductase for the efficient synthesis of an (*R*)-α-lipoic acid precursor. Adv. Synth. Catal..

[16] Chavan S.P., Praveen C., Ramakrishna G., Kalkote U. (2004). Enantioselective synthesis of *R*-(+)-α and *S*-(−)-α-lipoic acid. Tetrahedron Lett..

[17] Chen L.X., Wang X.Y., Lu W.H., Wu X.Q., Li J.H. (2016). Molecular imprinting: Perspectives and applications. Chem. Soc. Rev..

[18] Ji W., Ma X., Zhang J., Xie H., Liu F., Wang X. (2015). Preparation of the high purity gingerols from ginger by dummy molecularly imprinted polymers. J. Chromatogr. A.

[19] Ji W., Chen L., Ma X., Wang X., Gao Q., Geng Y., Huang L. (2014). Molecularly imprinted polymers with novel functional monomer for selective solid-phase extraction of gastrodin from the aqueous extract of *Gastrodia elata*. J. Chromatogr. A.

[20] Theodoridis G., Lasakova M., Skerikova V., Tegou A., Giantsiou N., Jandera P. (2006). Molecular imprinting of natural flavonoid antioxidants: Application in solid-phase extraction for the sample pretreatment of natural products prior to HPLC analysis. J. Sep. Sci..

[21] Zeng H., Wang Y.Z., Liu X.J., Kong J.H., Nie C. (2012). Preparation of molecular imprinted polymers using bi-functional monomer and bi-crosslinker for solid-phase extraction of rutin. Talanta.

[22] Chen L.X., Xu S.F., Li J.H. (2011). Recent advances in molecular imprinting technology: Current status, challenges and highlighted applications. Chem. Soc. Rev..

[23] Xu L., Huang Y.-A., Zhu Q.-J., Ye C. (2015). Chitosan in molecularly-imprinted polymers: Current and future prospects. Int. J. Mol. Sci..

[24] Xu L., Huang Y.-A., Zhu Q.-J., Ye C. (2016). Preparation and application of molecularly imprinted polymers based on chitosan. Chem. Ind. Eng. Prog..

[25] Yu Q., Deng S., Yu G. (2008). Selective removal of perfluorooctane sulfonate from aqueous solution using chitosan-based molecularly imprinted polymer adsorbents. Water Res..

[26] Liu B., Wang D., Li H., Xu Y., Zhang L. (2011). As(III) removal from aqueous solution using α-Fe2O3 impregnated chitosan beads with As(III) as imprinted ions. Desalination.

[27] Gupta K.C., Jabrail F.H. (2006). Glutaraldehyde and glyoxal cross-linked chitosan microspheres for controlled delivery of centchroman. Carbohydr. Res..

[28] Monier M., Ayad D., Wei Y., Sarhan A. (2010). Preparation of cross-linked chitosan/glyoxal molecularly imprinted resin for efficient chiral resolution of aspartic acid isomers. Biochem. Eng. J..

[29] Wu H., Zhao Y.-Y., Yu Y.-X., Jiang Z.-Y. (2007). Molecularly imprinted chitosan membrane for chiral resolution of phenylalanine isomers. J. Funct. Polym..

[30] Ogunlaja A.S., Coombes M.J., Torto N., Tshentu Z.R. (2014). The adsorptive extraction of oxidized sulfur-containing compounds from fuels by using molecularly imprinted chitosan materials. React. Funct. Polym..

[31] Wang Y., Wang E., Wu Z., Li H., Zhu Z., Zhu X., Dong Y. (2014). Synthesis of chitosan molecularly imprinted polymers for solid-phase extraction of methandrostenolone. Carbohydr. Polym..

[32] Ma X., Chen R., Zheng X., Youn H., Chen Z. (2011). Preparation of molecularly imprinted CS membrane for recognizing naringin in aqueous media. Polym. Bull..

[33] Huang Y.A., Xu L., Yang B., Zhu Q. (2018). Self-assembly system of α-lipoic acid as an antioxidant and preparation of molecularly imprinted polymers for its selective adsorption. Food Science.

[34] Ho Y.-S., McKay G. (1999). Pseudo-second order model for sorption processes. Process Biochem..

[35] Chiou M., Li H. (2003). Adsorption behavior of reactive dye in aqueous solution on chemical cross-linked chitosan beads. Chemosphere.

[36] Wong Y., Szeto Y., Cheung W., McKay G. (2003). Equilibrium studies for acid dye adsorption onto chitosan. Langmuir.

[37] Shaikh H., Memon N., Khan H., Bhanger M., Nizamani S. (2012). Preparation and characterization of molecularly imprinted polymer for di (2-ethylhexyl) phthalate: Application to sample clean-up prior to gas chromatographic determination. J. Chromatogr. A.

[38] Kirschner K.N., Yongye A.B., Tschampel S.M., González-Outeiriño J., Daniels C.R., Foley B.L., Woods R.J. (2008). GLYCAM06: A generalizable biomolecular force field. Carbohydrates. J. Comput. Chem..

[39] Wang J., Wolf R.M., Caldwell J.W., Kollman P.A., Case D.A. (2004). Development and testing of a general amber force field. J. Comput. Chem..

[40] Frisch M.J., Trucks G.W., Schlegel H.B., Scuseria G.E., Robb M.A., Cheeseman J.R., Scalmani G., Barone V., Mennucci B., Petersson G.A. (2009). Gaussian 09, Revision D. 01.

[41] Jorgensen W.L., Chandrasekhar J., Madura J.D., Impey R.W., Klein M.L. (1983). Comparison of simple potential functions for simulating liquid water. J. Chem. Phys..

[42] Abraham M.J., Murtola T., Schulz R., Páll S., Smith J.C., Hess B., Lindahl E. (2015). GROMACS: High performance molecular simulations through multi-level parallelism from laptops to supercomputers. SoftwareX.

