# Supplementary material for: Preparation of Chitosan Molecularly Imprinted Polymers and the Recognition Mechanism for Adsorption of Alpha-Lipoic Acid
Source: Molecules. 2020 Jan 13;25(2):312. doi: 10.3390/molecules25020312 (PMC7024251; doi:10.3390/molecules25020312)
Supplement: Supplementary file 1 [file molecules-25-00312-s001.pdf]

molecules

Supplemental Information

Preparation of Chitosan Molecularly Imprinted Polymers and the Recognition Mechanism for Adsorption of Alpha-Lipoic Acid

Long Xu [1,2], Ze-Xin Zhao [3], Yun-An Huang [4], Qiu-Jin Zhu [1,5*]

[1] School of Liquor and Food Engineering, Guizhou University, Guiyang 550025, China; xulong19891026@163.com
[2] School of Food Science and Engineering, South China University of Technology, Guangzhou 510640, China;
[3] School of Bioscience and Bioengineering, South China University of Technology, Guangzhou 510006, China; bizexin-zhao@mail.scut.edu.cn
[4] Department of Food Engineering, Guizhou Vocational College of Foodstuff Engineering, Guiyang 551400, China; hya127@163.com
[5] Key Laboratory of Agricultural and Animal Products Store and Processing of Guizhou Province, Guiyang 550025, China.
[*]Correspondence: ls.qjzhu@gzu.edu.cn; Tel.: +86-851-8385-1195

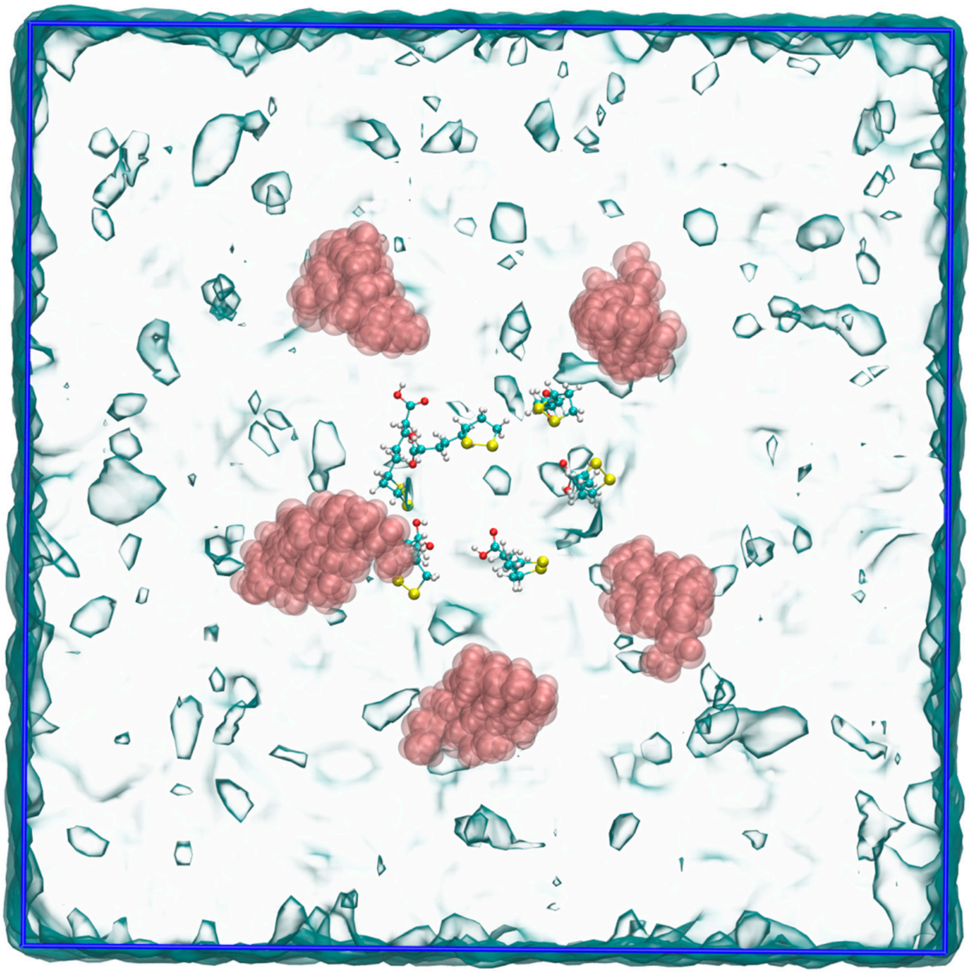

Figure S1. The initial model of ALAs (ball-and-stick model) and chitosan hexamers (vdW surface model) in water box.

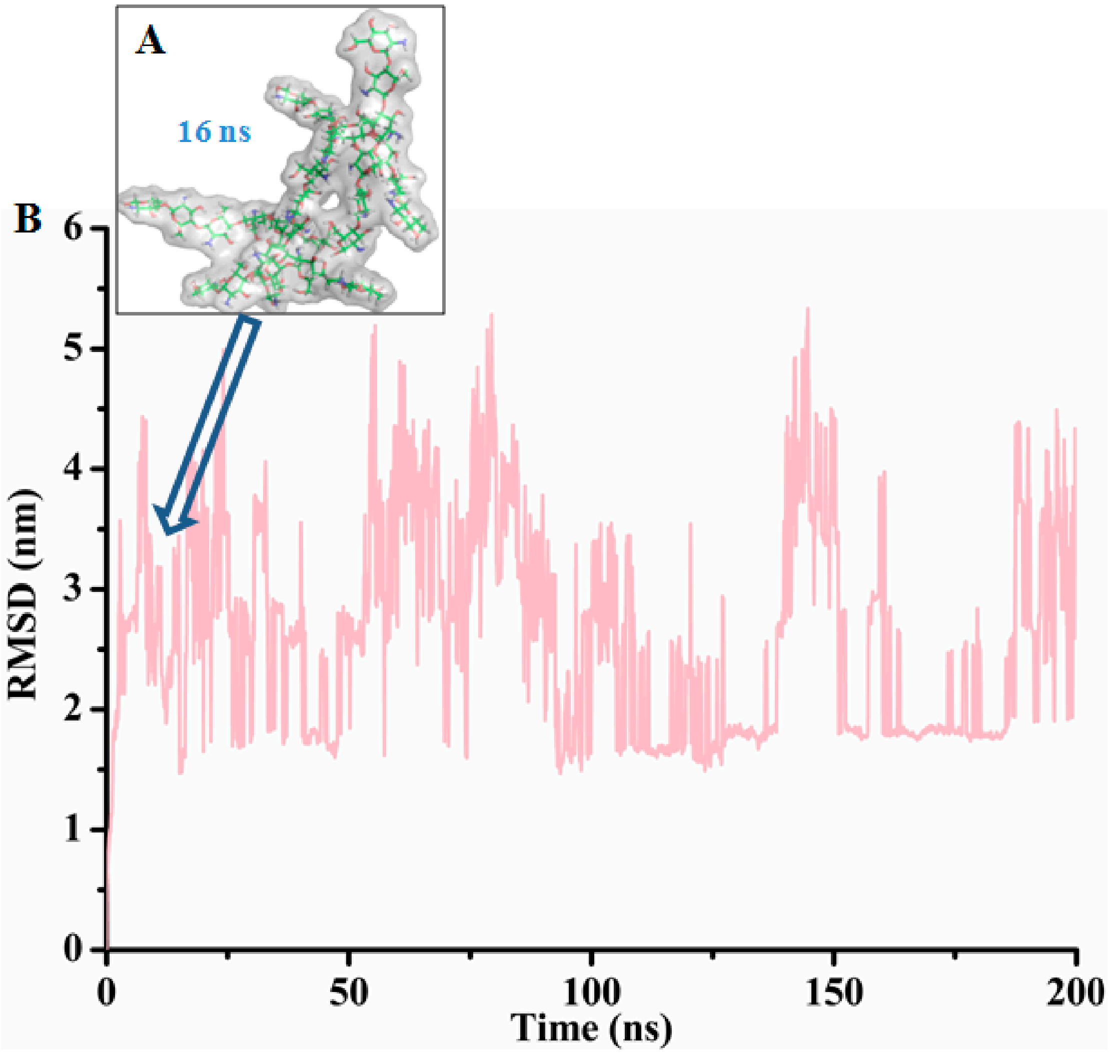

Figure S2. RMSD analysis. (**A**) The chitosan micelle model formed after 16 ns simulation time, (**B**) The RMSD curve of chitosan.
